# 5′-Methyl-4′-oxo-7′-phenyl-3′,4′-dihydro-1′*H*-spiro­[cyclo­hexane-1,2′-quinazoline]-8′-carbonitrile

**DOI:** 10.1107/S160053681102188X

**Published:** 2011-06-18

**Authors:** Jianhong Tang, Daxin Shi, Liupan Yan, Xuan Liu, Jiarong Li

**Affiliations:** a School of Chemical Engineering and Environment, Beijing Institute of Technology, Beijing 100081, People’s Republic of China; b College of Chemical Engineering, Huaqiao University, Xiamen Fujian 362021, People’s Republic of China

## Abstract

The title compound, C_21_H_21_N_3_O, was obtained by cyclo­condensation of 3-amino-5-methyl-[1,1′-biphen­yl]-2,4-di­car­bo­nitrile with cyclo­hexa­none. The six-membered 1,3-diaza ring assumes an envelope conformation [with the flap atom displaced by 0.511 (7) Å from the plane through the other ring atoms] and the cyclo­hexane ring displays a chair conformation.  The dihedral angle between the aromatic rings is 42.61 (7)°.In the crystal, the mol­ecules form hydrogen-bonded bands along [011].

## Related literature

For the medicinal and biological properities of dihydro­quinazolin-4(3*H*)-one derivatives, see: Deng *et al.* (2000 ▶); Chenard *et al.* (2000 ▶); Bertrand *et al.* (2001 ▶); Welch *et al.* (2001 ▶). For a related structure, see Zhang *et al.* (2008 ▶)
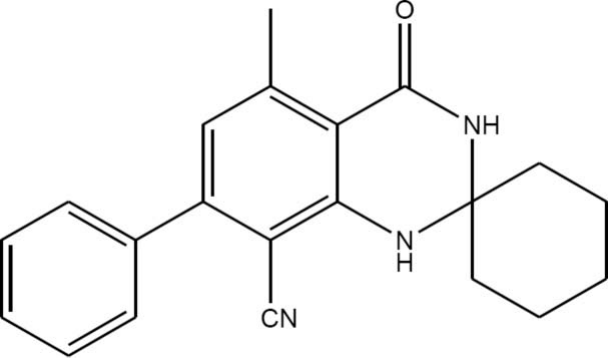


## Experimental

### 

#### Crystal data

C_21_H_21_N_3_O
*M* *_r_* = 331.41
Triclinic, 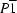

*a* = 7.1824 (15) Å
*b* = 11.233 (3) Å
*c* = 11.430 (3) Å
α = 101.858 (8)°
β = 93.794 (9)°
γ = 104.606 (9)°
*V* = 866.6 (4) Å^3^
*Z* = 2
Mo *K*α radiation
μ = 0.08 mm^−1^
*T* = 153 K
0.35 × 0.27 × 0.27 mm

#### Data collection

Rigaku AFC10/Saturn724+ diffractometer
9387 measured reflections
4521 independent reflections
3511 reflections with *I* > 2σ(*I*)
*R*_int_ = 0.024

#### Refinement

*R*[*F* ^2^ > 2σ(*F* ^2^)] = 0.048
*wR*(*F* ^2^) = 0.117
*S* = 1.00
4521 reflections
235 parameters
H atoms treated by a mixture of independent and constrained refinement
Δρ_max_ = 0.35 e Å^−3^
Δρ_min_ = −0.20 e Å^−3^

### 

Data collection: *CrystalClear* (Rigaku, 2004 ▶); cell refinement: *CrystalClear*; data reduction: *CrystalClear*; program(s) used to solve structure: *SHELXS97* (Sheldrick, 2008 ▶); program(s) used to refine structure: *SHELXL97* (Sheldrick, 2008 ▶); molecular graphics: *SHELXTL* (Sheldrick, 2008 ▶); software used to prepare material for publication: *SHELXL97*.

## Supplementary Material

Crystal structure: contains datablock(s) I, global. DOI: 10.1107/S160053681102188X/ld2014sup1.cif

Structure factors: contains datablock(s) I. DOI: 10.1107/S160053681102188X/ld2014Isup2.hkl

Supplementary material file. DOI: 10.1107/S160053681102188X/ld2014Isup3.cml

Additional supplementary materials:  crystallographic information; 3D view; checkCIF report

## Figures and Tables

**Table 1:** Hydrogen-bond geometry (Å, °)

*D*—H⋯*A*	*D*—H	H⋯*A*	*D*⋯*A*	*D*—H⋯*A*
N2—H2*N*⋯Ol^i^	0.920 (17)	1.998 (14)	2.9010 (17)	171.68 (14)
N1—H1*N*⋯N3^ii^	0.875 (18)	2.281 (14)	3.1188 (19)	160.24 (15)

Symmetry codes: (i) 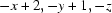; (ii) 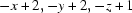.

**Fig. 1.**
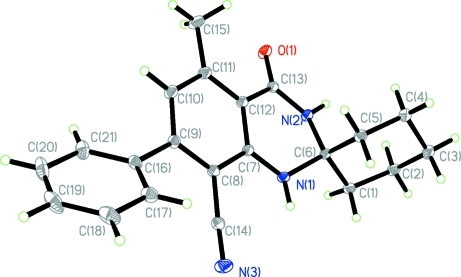
The molecular structure of (I) drawn with 50% probability ellipsoids

**Fig. 2.**
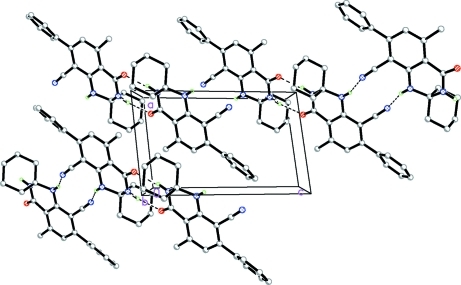
The crystal structure of (I) viewed along x axis

## supplementary crystallographic information

### Comment

The molecular structure of (I) is represented on Fig. 1. The 1,3-diaza ring exists in an envelope conformation with its C6 atom displaced by 0.511 (7)Å from the rest of the atoms of the ring (planar within 0.025 (1) Å). The geometry of the fused rings compares well with the related spiro[cyclopentane-1,2'(1'H)-quinazolin]-4'(3')-one] (Zhang *et al.*, 2008). The crystal packing is stabilized by intermolecular N—H···O and N—H···N hydrogen bonds forming alternating 8- and 12-membered centrosymmetric rings along [0 1 1] direction.

### Experimental

3-Amino-5-methyl-biphenyl-2,4-dicarbonitrile (0.1 mmol) was added to a mixture of cyclohexanone (3 ml) and sodium hydroxide (0.03 mmol). The reagents were heated at 373 K for 1.5 h. The reaction mixture was cooled to room temperature with water (15 ml) and then filtered to give the title compound. The product was recrystallizated from a mixed solvent (ethanol:petroleum ether 1:3) to give pale-yellow crystals.

M.p. 572–573 K. Spectra data: IR (KBr): 3329, 3067, 2934, 2221,1660, 1560, 1403, 697 cm^-1^; ^1^H-NMR(DMSO, p.p.m.):1.34–1.85 (10*H*, m, C_5_H_10_), 2.64 (3*H*, d, J = 1.4 Hz, CH_3_), 6.42(1*H*, s, NH), 6.71 (1*H*, s, ArH), 7.49–7.53 (5*H*, m, ArH), 8.26 (1*H*, s, NH); ESI-MS m/*z*: [*M*+H]^+^ 332.1; C_21_H_21_N_3_O:calcd. C 76.11, H 6.39, N 12.68; found C 76.12, H 6.55, N 12.06.

### Refinement

C—H bonds were refined within a riding/rotating model approximation with C—H distances 0.95–0.99 Å, and with *U*_iso_(H)=1.2*U*_eq_(C) or 1.5*U*_eq_(*C*)(methyl). H atoms of NH group were located in difference Fourrier syntheses and refined freely.

### Crystal data

**Table:**

C_21_H_21_N_3_O	*Z* = 2
*M**_r_* = 331.41	*F*(000) = 352
Triclinic, *P*1	*D*_x_ = 1.270 Mg m^−^^3^
Hall symbol: -P 1	Mo *K*α radiation, λ = 0.71073 Å
*a* = 7.1824 (15) Å	Cell parameters from 3260 reflections
*b* = 11.233 (3) Å	θ = 3.0–29.1°
*c* = 11.430 (3) Å	µ = 0.08 mm^−^^1^
α = 101.858 (8)°	*T* = 153 K
β = 93.794 (9)°	Block, colorless
γ = 104.606 (9)°	0.35 × 0.27 × 0.27 mm
*V* = 866.6 (4) Å^3^	

### Data collection

**Table:**

Rigaku AFC10/Saturn724+ diffractometer	3511 reflections with *I* > 2σ(*I*)
Radiation source: rotating anode	*R*_int_ = 0.024
graphite	θ_max_ = 29.1°, θ_min_ = 3.0°
Detector resolution: 28.5714 pixels mm^-1^	*h* = −9→7
phi and ω scans	*k* = −15→15
9387 measured reflections	*l* = −15→15
4521 independent reflections	

### Refinement

**Table:**

Refinement on *F*^2^	Primary atom site location: structure-invariant direct methods
Least-squares matrix: full	Secondary atom site location: difference Fourier map
*R*[*F*^2^ > 2σ(*F*^2^)] = 0.048	Hydrogen site location: inferred from neighbouring sites
*wR*(*F*^2^) = 0.117	H atoms treated by a mixture of independent and constrained refinement
*S* = 1.00	*w* = 1/[σ^2^(*F*_o_^2^) + (0.0516*P*)^2^ + 0.259*P*] where *P* = (*F*_o_^2^ + 2*F*_c_^2^)/3
4521 reflections	(Δ/σ)_max_ < 0.001
235 parameters	Δρ_max_ = 0.35 e Å^−^^3^
0 restraints	Δρ_min_ = −0.20 e Å^−^^3^

### Special details

**Table:**

Geometry. All e.s.d.'s (except the e.s.d. in the dihedral angle between two l.s. planes) are estimated using the full covariance matrix. The cell e.s.d.'s are taken into account individually in the estimation of e.s.d.'s in distances, angles and torsion angles; correlations between e.s.d.'s in cell parameters are only used when they are defined by crystal symmetry. An approximate (isotropic) treatment of cell e.s.d.'s is used for estimating e.s.d.'s involving l.s. planes.
Refinement. Refinement of *F*^2^ against ALL reflections. The weighted *R*-factor *wR* and goodness of fit *S* are based on *F*^2^, conventional *R*-factors *R* are based on *F*, with *F* set to zero for negative *F*^2^. The threshold expression of *F*^2^ > σ(*F*^2^) is used only for calculating *R*-factors(gt) *etc*. and is not relevant to the choice of reflections for refinement. *R*-factors based on *F*^2^ are statistically about twice as large as those based on *F*, and *R*- factors based on ALL data will be even larger.

### Fractional atomic coordinates and isotropic or equivalent isotropic displacement parameters (Å^2^)

**Table:**

	*x*	*y*	*z*	*U*_iso_*/*U*_eq_	
O1	0.79935 (15)	0.44593 (8)	0.06825 (9)	0.0303 (2)	
N1	0.97094 (15)	0.77778 (10)	0.30982 (9)	0.0212 (2)	
N2	1.00920 (15)	0.63845 (9)	0.13608 (9)	0.0201 (2)	
N3	0.84792 (18)	0.93552 (12)	0.57411 (11)	0.0336 (3)	
C1	1.26985 (18)	0.83252 (12)	0.22053 (11)	0.0212 (3)	
H1A	1.3292	0.7806	0.2637	0.025*	
H1B	1.2955	0.9177	0.2742	0.025*	
C2	1.36630 (19)	0.84416 (12)	0.10637 (12)	0.0250 (3)	
H2A	1.5044	0.8920	0.1293	0.030*	
H2B	1.3612	0.7587	0.0593	0.030*	
C3	1.2676 (2)	0.91080 (13)	0.02799 (13)	0.0290 (3)	
H3A	1.3260	0.9095	−0.0482	0.035*	
H3B	1.2883	1.0002	0.0704	0.035*	
C4	1.0504 (2)	0.84591 (13)	−0.00007 (12)	0.0255 (3)	
H4A	1.0296	0.7585	−0.0479	0.031*	
H4B	0.9881	0.8920	−0.0488	0.031*	
C5	0.95716 (18)	0.84267 (11)	0.11605 (11)	0.0214 (3)	
H5A	0.8168	0.7998	0.0958	0.026*	
H5B	0.9719	0.9302	0.1617	0.026*	
C6	1.05070 (17)	0.77279 (11)	0.19470 (10)	0.0179 (2)	
C7	0.78933 (17)	0.70442 (11)	0.31283 (10)	0.0185 (2)	
C8	0.68158 (18)	0.73621 (11)	0.40828 (11)	0.0193 (2)	
C9	0.49368 (18)	0.66095 (12)	0.41115 (11)	0.0203 (2)	
C10	0.41853 (19)	0.55284 (12)	0.31972 (12)	0.0242 (3)	
H10	0.2911	0.5021	0.3205	0.029*	
C11	0.52333 (19)	0.51594 (12)	0.22663 (12)	0.0234 (3)	
C12	0.71034 (18)	0.59166 (11)	0.22332 (10)	0.0190 (2)	
C13	0.83918 (18)	0.55304 (11)	0.13493 (11)	0.0203 (2)	
C14	0.77055 (18)	0.84636 (12)	0.50192 (11)	0.0224 (3)	
C15	0.4289 (2)	0.39423 (14)	0.13529 (14)	0.0364 (4)	
H15A	0.4901	0.3288	0.1491	0.044*	
H15B	0.4446	0.4074	0.0540	0.044*	
H15C	0.2905	0.3674	0.1434	0.044*	
C16	0.37353 (18)	0.69620 (13)	0.50673 (11)	0.0228 (3)	
C17	0.3629 (2)	0.82022 (14)	0.54351 (12)	0.0281 (3)	
H17	0.4364	0.8841	0.5090	0.034*	
C18	0.2455 (2)	0.85120 (17)	0.63036 (13)	0.0377 (4)	
H18	0.2394	0.9361	0.6553	0.045*	
C19	0.1375 (2)	0.75827 (19)	0.68029 (14)	0.0431 (4)	
H19	0.0572	0.7793	0.7396	0.052*	
C20	0.1462 (2)	0.63526 (18)	0.64427 (14)	0.0413 (4)	
H20	0.0718	0.5717	0.6789	0.050*	
C21	0.2633 (2)	0.60356 (15)	0.55756 (13)	0.0314 (3)	
H21	0.2681	0.5184	0.5329	0.038*	
H2N	1.081 (2)	0.6152 (15)	0.0762 (15)	0.034 (4)*	
H1N	1.012 (3)	0.8526 (17)	0.3587 (16)	0.039 (5)*	

### Atomic displacement parameters (Å^2^)

**Table:**

	*U*^11^	*U*^22^	*U*^33^	*U*^12^	*U*^13^	*U*^23^
O1	0.0370 (6)	0.0169 (4)	0.0291 (5)	−0.0006 (4)	0.0161 (4)	−0.0058 (4)
N1	0.0228 (5)	0.0177 (5)	0.0172 (5)	0.0004 (4)	0.0073 (4)	−0.0041 (4)
N2	0.0229 (5)	0.0151 (5)	0.0203 (5)	0.0039 (4)	0.0092 (4)	−0.0010 (4)
N3	0.0293 (6)	0.0297 (6)	0.0331 (6)	0.0038 (5)	0.0098 (5)	−0.0087 (5)
C1	0.0185 (6)	0.0199 (6)	0.0211 (6)	0.0024 (5)	0.0036 (5)	−0.0010 (5)
C2	0.0197 (6)	0.0228 (6)	0.0266 (6)	0.0001 (5)	0.0080 (5)	−0.0024 (5)
C3	0.0287 (7)	0.0260 (7)	0.0285 (7)	−0.0013 (5)	0.0099 (5)	0.0064 (5)
C4	0.0276 (7)	0.0241 (6)	0.0227 (6)	0.0030 (5)	0.0041 (5)	0.0057 (5)
C5	0.0204 (6)	0.0178 (6)	0.0239 (6)	0.0038 (5)	0.0047 (5)	0.0014 (5)
C6	0.0193 (6)	0.0145 (5)	0.0170 (5)	0.0024 (4)	0.0060 (4)	−0.0012 (4)
C7	0.0210 (6)	0.0158 (5)	0.0173 (5)	0.0041 (4)	0.0050 (4)	0.0012 (4)
C8	0.0218 (6)	0.0174 (5)	0.0176 (5)	0.0055 (5)	0.0060 (4)	0.0000 (4)
C9	0.0210 (6)	0.0206 (6)	0.0204 (6)	0.0068 (5)	0.0066 (5)	0.0046 (5)
C10	0.0187 (6)	0.0232 (6)	0.0270 (6)	0.0015 (5)	0.0062 (5)	0.0015 (5)
C11	0.0230 (6)	0.0201 (6)	0.0230 (6)	0.0029 (5)	0.0052 (5)	−0.0009 (5)
C12	0.0218 (6)	0.0158 (5)	0.0172 (5)	0.0034 (5)	0.0050 (4)	0.0000 (4)
C13	0.0250 (6)	0.0167 (5)	0.0169 (5)	0.0036 (5)	0.0059 (5)	−0.0001 (4)
C14	0.0220 (6)	0.0233 (6)	0.0212 (6)	0.0067 (5)	0.0091 (5)	0.0009 (5)
C15	0.0278 (7)	0.0291 (7)	0.0365 (8)	−0.0064 (6)	0.0093 (6)	−0.0113 (6)
C16	0.0190 (6)	0.0307 (7)	0.0190 (6)	0.0080 (5)	0.0051 (5)	0.0039 (5)
C17	0.0264 (7)	0.0339 (7)	0.0236 (6)	0.0112 (6)	0.0058 (5)	0.0013 (5)
C18	0.0364 (8)	0.0506 (9)	0.0276 (7)	0.0229 (7)	0.0071 (6)	−0.0024 (7)
C19	0.0370 (9)	0.0760 (13)	0.0255 (7)	0.0292 (9)	0.0149 (6)	0.0114 (8)
C20	0.0318 (8)	0.0683 (12)	0.0357 (8)	0.0184 (8)	0.0173 (7)	0.0278 (8)
C21	0.0271 (7)	0.0408 (8)	0.0325 (7)	0.0123 (6)	0.0112 (6)	0.0158 (6)

### Geometric parameters (Å, °)

**Table:**

O1—C13	1.2374 (14)	C7—C8	1.4169 (15)
N1—C7	1.3628 (16)	C8—C9	1.4068 (17)
N1—C6	1.4658 (15)	C8—C14	1.4323 (17)
N1—H1N	0.875 (18)	C9—C10	1.3898 (17)
N2—C13	1.3468 (16)	C9—C16	1.4881 (16)
N2—C6	1.4650 (15)	C10—C11	1.3974 (17)
N2—H2N	0.920 (17)	C10—H10	0.9500
N3—C14	1.1453 (17)	C11—C12	1.4045 (17)
C1—C6	1.5295 (17)	C11—C15	1.5071 (18)
C1—C2	1.5310 (17)	C12—C13	1.4870 (16)
C1—H1A	0.9900	C15—H15A	0.9800
C1—H1B	0.9900	C15—H15B	0.9800
C2—C3	1.525 (2)	C15—H15C	0.9800
C2—H2A	0.9900	C16—C17	1.3931 (19)
C2—H2B	0.9900	C16—C21	1.3933 (19)
C3—C4	1.5268 (19)	C17—C18	1.3909 (18)
C3—H3A	0.9900	C17—H17	0.9500
C3—H3B	0.9900	C18—C19	1.383 (3)
C4—C5	1.5288 (17)	C18—H18	0.9500
C4—H4A	0.9900	C19—C20	1.377 (3)
C4—H4B	0.9900	C19—H19	0.9500
C5—C6	1.5335 (18)	C20—C21	1.3901 (19)
C5—H5A	0.9900	C20—H20	0.9500
C5—H5B	0.9900	C21—H21	0.9500
C7—C12	1.4147 (16)		
			
C7—N1—C6	119.48 (10)	C12—C7—C8	119.21 (11)
C7—N1—H1N	119.5 (11)	C9—C8—C7	120.81 (11)
C6—N1—H1N	112.5 (11)	C9—C8—C14	121.21 (10)
C13—N2—C6	123.65 (10)	C7—C8—C14	117.97 (11)
C13—N2—H2N	114.8 (10)	C10—C9—C8	118.30 (11)
C6—N2—H2N	118.2 (10)	C10—C9—C16	119.88 (11)
C6—C1—C2	113.08 (10)	C8—C9—C16	121.80 (11)
C6—C1—H1A	109.0	C9—C10—C11	122.48 (12)
C2—C1—H1A	109.0	C9—C10—H10	118.8
C6—C1—H1B	109.0	C11—C10—H10	118.8
C2—C1—H1B	109.0	C10—C11—C12	119.18 (11)
H1A—C1—H1B	107.8	C10—C11—C15	117.49 (12)
C3—C2—C1	111.97 (11)	C12—C11—C15	123.33 (11)
C3—C2—H2A	109.2	C11—C12—C7	119.93 (10)
C1—C2—H2A	109.2	C11—C12—C13	122.62 (10)
C3—C2—H2B	109.2	C7—C12—C13	117.22 (11)
C1—C2—H2B	109.2	O1—C13—N2	121.02 (11)
H2A—C2—H2B	107.9	O1—C13—C12	122.42 (11)
C2—C3—C4	110.60 (11)	N2—C13—C12	116.43 (10)
C2—C3—H3A	109.5	N3—C14—C8	177.19 (13)
C4—C3—H3A	109.5	C11—C15—H15A	109.5
C2—C3—H3B	109.5	C11—C15—H15B	109.5
C4—C3—H3B	109.5	H15A—C15—H15B	109.5
H3A—C3—H3B	108.1	C11—C15—H15C	109.5
C3—C4—C5	110.76 (11)	H15A—C15—H15C	109.5
C3—C4—H4A	109.5	H15B—C15—H15C	109.5
C5—C4—H4A	109.5	C17—C16—C21	118.90 (12)
C3—C4—H4B	109.5	C17—C16—C9	121.09 (12)
C5—C4—H4B	109.5	C21—C16—C9	119.96 (12)
H4A—C4—H4B	108.1	C18—C17—C16	120.52 (14)
C4—C5—C6	111.17 (10)	C18—C17—H17	119.7
C4—C5—H5A	109.4	C16—C17—H17	119.7
C6—C5—H5A	109.4	C19—C18—C17	119.88 (15)
C4—C5—H5B	109.4	C19—C18—H18	120.1
C6—C5—H5B	109.4	C17—C18—H18	120.1
H5A—C5—H5B	108.0	C20—C19—C18	120.13 (13)
N2—C6—N1	106.03 (9)	C20—C19—H19	119.9
N2—C6—C1	110.11 (9)	C18—C19—H19	119.9
N1—C6—C1	108.50 (10)	C19—C20—C21	120.33 (15)
N2—C6—C5	110.77 (10)	C19—C20—H20	119.8
N1—C6—C5	111.09 (10)	C21—C20—H20	119.8
C1—C6—C5	110.24 (10)	C20—C21—C16	120.24 (15)
N1—C7—C12	119.96 (10)	C20—C21—H21	119.9
N1—C7—C8	120.76 (10)	C16—C21—H21	119.9
			
C6—C1—C2—C3	52.46 (14)	C9—C10—C11—C15	177.78 (13)
C1—C2—C3—C4	−53.99 (14)	C10—C11—C12—C7	−0.69 (19)
C2—C3—C4—C5	57.35 (14)	C15—C11—C12—C7	−179.68 (13)
C3—C4—C5—C6	−58.74 (14)	C10—C11—C12—C13	173.71 (12)
C13—N2—C6—N1	41.95 (15)	C15—C11—C12—C13	−5.3 (2)
C13—N2—C6—C1	159.13 (11)	N1—C7—C12—C11	179.85 (12)
C13—N2—C6—C5	−78.67 (14)	C8—C7—C12—C11	3.05 (18)
C7—N1—C6—N2	−45.90 (14)	N1—C7—C12—C13	5.16 (17)
C7—N1—C6—C1	−164.16 (10)	C8—C7—C12—C13	−171.65 (11)
C7—N1—C6—C5	74.51 (14)	C6—N2—C13—O1	168.01 (12)
C2—C1—C6—N2	69.94 (13)	C6—N2—C13—C12	−16.04 (17)
C2—C1—C6—N1	−174.42 (10)	C11—C12—C13—O1	−8.7 (2)
C2—C1—C6—C5	−52.57 (13)	C7—C12—C13—O1	165.83 (12)
C4—C5—C6—N2	−66.58 (13)	C11—C12—C13—N2	175.40 (12)
C4—C5—C6—N1	175.86 (10)	C7—C12—C13—N2	−10.05 (17)
C4—C5—C6—C1	55.55 (13)	C9—C8—C14—N3	−174 (3)
C6—N1—C7—C12	25.46 (17)	C7—C8—C14—N3	6(3)
C6—N1—C7—C8	−157.79 (11)	C10—C9—C16—C17	−134.73 (14)
N1—C7—C8—C9	179.65 (11)	C8—C9—C16—C17	43.49 (19)
C12—C7—C8—C9	−3.57 (18)	C10—C9—C16—C21	42.70 (18)
N1—C7—C8—C14	−0.92 (18)	C8—C9—C16—C21	−139.07 (14)
C12—C7—C8—C14	175.86 (11)	C21—C16—C17—C18	0.6 (2)
C7—C8—C9—C10	1.68 (18)	C9—C16—C17—C18	178.05 (13)
C14—C8—C9—C10	−177.73 (12)	C16—C17—C18—C19	−0.3 (2)
C7—C8—C9—C16	−176.57 (12)	C17—C18—C19—C20	0.1 (2)
C14—C8—C9—C16	4.01 (19)	C18—C19—C20—C21	0.0 (3)
C8—C9—C10—C11	0.8 (2)	C19—C20—C21—C16	0.3 (2)
C16—C9—C10—C11	179.05 (12)	C17—C16—C21—C20	−0.6 (2)
C9—C10—C11—C12	−1.3 (2)	C9—C16—C21—C20	−178.06 (14)

### Hydrogen-bond geometry (Å, °)

**Table:**

*D*—H···*A*	*D*—H	H···*A*	*D*···*A*	*D*—H···*A*
N2—H2N···Ol^i^	0.920 (17)	1.998 (14)	2.9010 (17)	171.68 (14)
N1—H1N···N3^ii^	0.875 (18)	2.281 (14)	3.1188 (19)	160.24 (15)

Symmetry codes: (i) −*x*+2, −*y*+1, −*z*; (ii) −*x*+2, −*y*+2, −*z*+1.

## References

[bb1] Bertrand, L. C., Welch, W. M., Blake, J. F., Butler, T. W., Reinhold, A., Ewing, F. E., Menniti, F. S. & Pagnozzi, M. J. (2001). *J. Med. Chem.* **44**, 1710–1717. 10.1021/jm000522p 11356106

[bb2] Chenard, B. L., Menniti, F. S., Pagnozzi, M. J., Shenk, K. D., Ewing, F. E. & Welch, W. M. (2000). *Bioorg. Med. Chem. Lett.* **10**, 1203–1205. 10.1016/s0960-894x(00)00216-x 10866381

[bb3] Deng, Y. H., Yang, R. S. & Yang, Y. (2000). *Chin. Pharm. Sci.* **9**, 116–118.

[bb4] Rigaku (2004). *CrystalClear* Rigaku Corporation, Tokyo, Japan.

[bb5] Sheldrick, G. M. (2008). *Acta Cryst.* A**64**, 112–122. 10.1107/S0108767307043930 18156677

[bb6] Welch, W. M., Ewing, F. E., Huang, J., Menniti, F. S., Pagnozzi, M. J., Kelly, K., Seymour, P. A., Guanowsky, V., Guhan, S., Guinn, M. R., Critchett, D., Lazzaro, J., Ganong, A. H., Devries, K. M., Staigers, T. L. & Chenard, B. L. (2001). *Bioorg. Med. Chem. Lett.* **11**, 177–181. 10.1016/s0960-894x(00)00622-3 11206453

[bb7] Zhang, L., Li, J., Shi, D. & Chen, J. (2008). *Acta Cryst.* E**64**, o449. 10.1107/S1600536807066706 PMC2960329 21201476

